# Open Source IIoT Solution for Gas Waste Monitoring in Smart Factory

**DOI:** 10.3390/s22082972

**Published:** 2022-04-13

**Authors:** Mark Waters, Pawel Waszczuk, Rodney Ayre, Alain Dreze, Don McGlinchey, Babakalli Alkali, Gordon Morison

**Affiliations:** 1 School of Computing, Engineering and Built Environment, Glasgow Caledonian University, 70 Cowcaddens Road, Glasgow G4 0BA, UK; mark.waters@gcu.ac.uk (M.W.); pawel.waszczuk@gcu.ac.uk (P.W.); d.mcglinchey@gcu.ac.uk (D.M.); babakalli.alkali@gcu.ac.uk (B.A.); 2 Mitsubishi Electric Air-Conditioning Systems Europe Ltd., Houston Industrial Estate, Livingston EH54 5EQ, UK; rodney.ayre@m-ace.mee.com (R.A.); alain.dreze@m-ace.mee.com (A.D.)

**Keywords:** Industry 4.0, IIoT, smart factory, smart manufacturing, RAMI 4.0, open source, waste monitoring

## Abstract

Rapid development of smart manufacturing techniques in recent years is influencing production facilities. Factories must both keep up with smart technologies as well as upskill their workforce to remain competitive. One of the recent concerns is how businesses can contribute to environmental sustainability and how to reduce operating costs. In this article authors present a method of measuring gas waste using Industrial Internet of Things (IIoT) sensors and open-source solutions utilised on a brownfield production asset. The article provides a result of an applied research initiative in a live manufacturing facility. The design followed the Reference Architectural Model for Industry 4.0 (RAMI 4.0) model to provide a coherent smart factory system. The presented solution’s goal is to provide factory supervisors with information about gas waste which is generated during the production process. To achieve this an operational technology (OT) network was installed and Key Performance Indicators (KPIs) dashboards were designed. Based on the information provided by the system, the business can be more aware of the production environment and can improve its efficiency.

## 1. Introduction

Modern industry is changing in a way never observed before. Due to the exploitation of digital technologies, manufacturing is becoming more customer oriented, efficient, productive, and resourceful [1,2]. The concept of the fourth industrial revolution (I4.0) has arisen to answer today’s industrial demands and expectations. The Internet of Things (IoT), big data, robotic systems, and additive manufacturing are just few of the pillars of the revolution [3]. An important role in this change is reserved for integration of the factory with the entire lifecycle of the product and supply chain. The key to enable I4.0 is the adoption of digital technologies, interconnectivity, data gathering and cybersecurity [4]. However, one cannot overlook the human factor. Existing competences and skills of shop floor personnel must be supplemented by the adoption of cutting-edge technologies [5]. The nature of management also needs to change; decision-making process must adapt to be data orientated, as access to real time information and Key Performance Indicators (KPIs) enable implementation of new business models [6].

**Figure 1:**
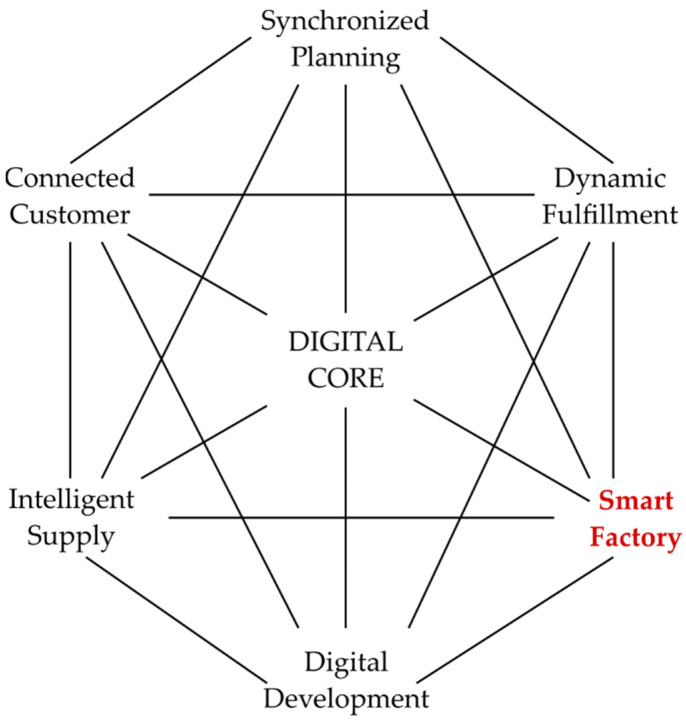
Digital supply chain network.

The concept of the Smart Factory (SF) is a very important part of the I4.0 revolution, as it combines physical machines and business processes (cyberphysical systems) using sophisticated techniques involving Artificial Intelligence (AI), statistical analysis and optimisation. It is envisaged as a flexible system that is able to adjust its performance reacting to its current environment. This is performed using collected production data and by the autonomous control of machinery [7]. Furthermore, SF is a part of the supply chain, where information from the shop floor can be used to enhance relationships with suppliers and customers [8]. Figure 1 shows an illustration of bodies taking part in the digital supply network and their interconnections. This diagram shows how each entity can generate and share information to adjust to a changing environment.

In recent years, a significant surge in investments in SF has been seen and according to Geissbauer et al. [9] nine out of the companies surveyed in 2017 were intending to increase spending on digital transformation. This drives a huge demand for the adoption of emerging technologies and for skilled, cross-disciplinary engineers. Petit et al. [10] state that a lack of employees with the required qualifications is seen as one of the major obstacles in the implementation of I4.0 technologies. One of the objectives of this article is to demonstrate how cooperation between universities and businesses can reduce the skill gap and boost the adoption of new technologies.

The research initiative was driven by M-ACE’s sustainability and cost reduction goals. In attempts to eliminate production waste and lower operating costs, M-ACE wished to investigate the use of consumables across the production facility. The process understudy was suspected to be inefficient due to its age and simplicity of the control system. The sensors installed onto the machine were able to provide the findings of this research but have multiple purposes for use in the production process. The data was also useful for maintenance, quality control and live production status. The installation of new sensors was limited to retrofitting as to not disrupt the continuous manufacturing process.

The work presented here focusses on installing a bespoke SF solution to a single process in a live production facility. The goal of the system is to first identify, and then reduce, inefficiencies in the process using data to inform operational decisions. The main contribution of this article is the design, implementation and integration of a data acquisition system for industrial purposes based on open-source solutions within an existing brownfield system. Best practices as well as common problems will be listed to facilitate replication of the presented solution.

This article is structured as follows, Section 2 provides a background about the subject facility and the reference architecture followed to implement SF. Section 3 describes the problem faced in the manufacturing process the SF solution aimed to tackle. Section 4 presents the installed solution, addressing the issues raised in the previous. Section 5 discusses the outcome of the research and how the solution feedback into the production process to implement real change. Finally, Section 6 provides a summary of the article.

## 2. Background

Mitsubishi Air Conditioning Europe (M-ACE) is an example of a manufacturer experiencing an engineering skills gap. In an aim to address this issue, a knowledge transfer partnership (KTP) with Glasgow Caledonian University (GCU) was established. The partnership aimed to help M-ACE research SF solutions bespoke to their needs and implement innovative projects in a short space of time.

The authors of this article were employed by GCU and placed in M-ACE to implement SF techniques to the manufacturing facility. Solutions were applied to a live production environment to install real change and business effectiveness. Due to the nature of the partnership, the authors had access to real data, assets and infrastructure used for the production of air conditioning units.

The strategy of deploying I4.0 technologies in a factory strongly depends on the condition of existing production equipment. In the case of greenfield, we are able to plan network infrastructure, level of automation, type of fieldbuses, etc.; however in this brownfield case, we were limited by the technologies already implemented [11]. It is common to find existing sites with varying proprietary equipment and field protocols, which are hard to integrate and network.

Outdated equipment requires retrofitting, which not only solves the problem of updating machines to communicate, but it also allows for upgrading the control software without disrupting production [12]. Heterogeneous assets create further challenges while designing SF roll out. The abundance of variety in device age and vendor, representing various levels of digital advancement causes integration issues. Manufacturing facilities invest in control equipment with the expectation of a long operating lifetime and Return on Investment (ROI). Legacy equipment will therefore always be present in an established factory and will need to be integrated to ensure SF compliance. To overcome this problem, industrial protocols such as Message Queuing Telemetry Transport (MQTT) and Open Platform Communication Unified Architecture (OPC UA) were developed. These protocols enable users to interconnect assets provided by different vendors, using various field protocols [13].

Poor network infrastructure is a further obstacle. With data gathering being the backbone of each SF, the supporting network should be able to handle the simultaneous transfer of a large amount of data [14]. A consideration when implementing the supporting network is whether to differentiate between the business IT equipment and the operational technology (OT) equipment. In most factories an IT network infrastructure will already exist, however building a separate network to isolate the two business functions would increase reliability and security [15,16]. Cybersecurity, in the context of SF is becoming a problem of upmost significance, where increasing the number of devices in a network make SFs susceptible to cyber-attacks [17]. Using certified, trustworthy cybersecurity providers, and offering proper separation of site networks from outside threats is therefore desired.

**Figure 2:**
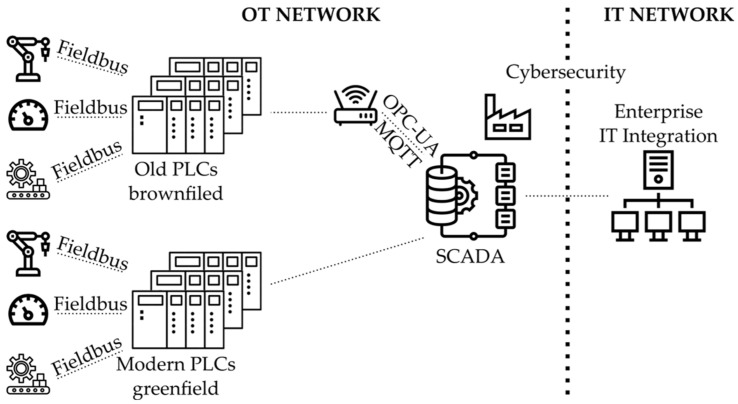
Data collection from operational technology (OT) to information technology IT networks. Programmable Logic Controller (PLC); Open Platform Communication Unified Architecture; Message Queuing Telemetry Transport (MQTT); Supervisory Control and Data Acquisition (SCADA).

Another prevalent issue with brownfield sites is missing or out of date documentation of industrial machines. Drawings of assets that are not properly maintained in configuration control make the replacement of Programmable Logic Controllers (PLC) or sensors much more labour intensive than retrofitting [18]. Figure 2 shows an example of connecting shop floors’ assets via old and modern PLCs to an enterprise network. This illustrates the flow of data from an OT network crossing a boundary through cybersecurity to the IT enterprise network.

**Figure 3:**
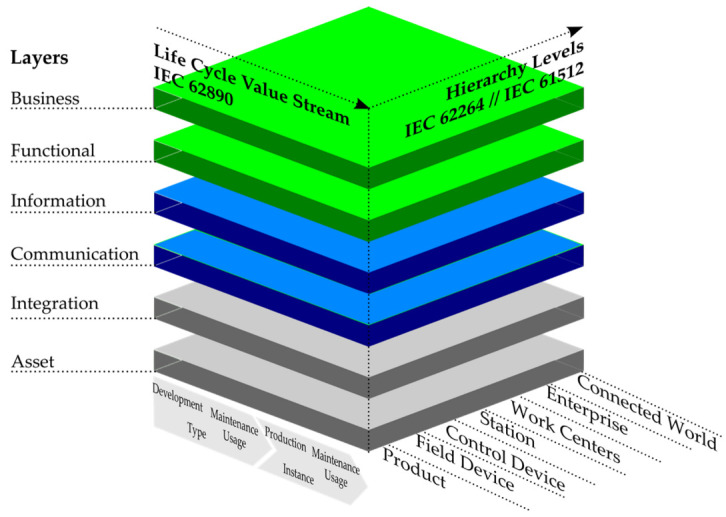
Reference Architectural Model for Industry 4.0 (RAMI 4.0). International Electrotechnical Commission (IEC).

The Reference Architectural Model for Industry 4.0 (RAMI 4.0) [19] was created to provide all of the stakeholders involved in I4.0 initiatives a common platform of understanding. RAMI 4.0 is a three-dimensional map showing how to approach the deployment of I4.0 in a structured manner. Companies using this framework as a guidance can design and develop future products utilising I4.0 standards. The RAMI 4.0 metamodel comprises of three axes: hierarchy levels, life cycle and value stream and layers. Figure 3 represents the RAMI 4.0 model.

The RAMI 4.0 model represents complex relationships between each element, breaking it down into smaller and simpler packages, which can be developed independently [20]. The “Hierarchy Levels” axis is an extended version of ANSI/ISA-95 standard, characterising connections between enterprise control systems and its other functions. The “Life Cycle Value Stream” axis represents the facilities and products life cycle, based on the IEC 62890 standard. The “Layers” axis comprises of six layers describing in a structured way how to break down properties of an individual machine or device. The RAMI 4.0 model aids the design of machines and processes, making sure they adhere to I4.0 canons. One of the purposes of this article is to present an implementation of a monitoring system based on open-source solutions utilising a RAMI 4.0 methodology. Each of the technologies were mapped onto a RAMI 4.0 model to visualise and validate how the implemented solution aligns with I4.0 standards. 

The rapid growth of smart manufacturing has resulted in an increase of interest in open-source solutions. Open Source Software (OSS) is often used to supplement existing internal software systems and to displace proprietary software when a more flexible solution is required [21]. Adoption of OSS involves no licensing costs and is free to use; therefore, the only cost involved is the human resource for implementation. Developed applications can be tailored according to the task in a relatively short time and low effort. OSS is easy to upgrade and integrate with other system as many libraries and Application Programming Interfaces (APIs) were created to bridge different systems. There is also an abundance of choice and cross platform support to suit most applications. Users are free to select one that suits best, e.g., programming language, operating system (OS), local/cloud, etc.

There is also an increase in usage of Open Source Hardware (OSH) in the manufacturing industries [22]. Devices such as Arduino or Raspberry Pi can be often found on the shop floor, particularly in an Internet of Things context. This type of hardware is used due to its low cost, ease of implementation and integration with OSS. The main drawbacks that are linked to OSH are its low robustness and lack of professional support. Nevertheless, many research publications regarding OSH utilisation can be found [23,24,25,26] which demonstrates its popularity. It is also worth noting that recently OSH is becoming more industry friendly; for example, Kunbus [27] is an Industrial Personal Computer (IPC) based on the Raspberry Pi. This solution introduces many industrial features such as DIN-rail mounting, rugged housing, and analogue and digital I/O modules. The operating system is Raspberry Pi-based, making OSS readily supported.

## 3. Problem Description

The main challenge faced when implementing SF solutions in the facility above all else was the production cycle. The production line operates at a near 24/7 rate with only a small number of hours set aside each fortnight for maintenance, which limited access to the equipment. In order to capture data, downtime from production was required to first investigate how the control system operated.

The machine under study for this research was a piece of legacy equipment, presenting further issues. The control system based on a legacy Mitsubishi PLC that was not designed with data gathering functions and providing only basic control. No modern ethernet connectivity was available on this model of PLC and data could therefore not be automatically collected over a network. Legacy equipment also introduced the problem of poor documentation and configuration control. Over the long years of service, the original wiring of the machine had been altered without any record, rendering the documentation inaccurate. The same issue presented itself in the ladder logic control program, where the original software had been overwritten and changed without configuration control. Moreover, comments describing variables used in the program were erased, making it even harder to decipher. 

All the above factors contributed to increasing the time and resources required for investigating the operation of the machine. Control signals were required to be checked via an oscilloscope to ensure their purpose and logic. All investigations could only be performed outside the scheduled production hours. 

### 3.1. Process Description

**Figure 4:**
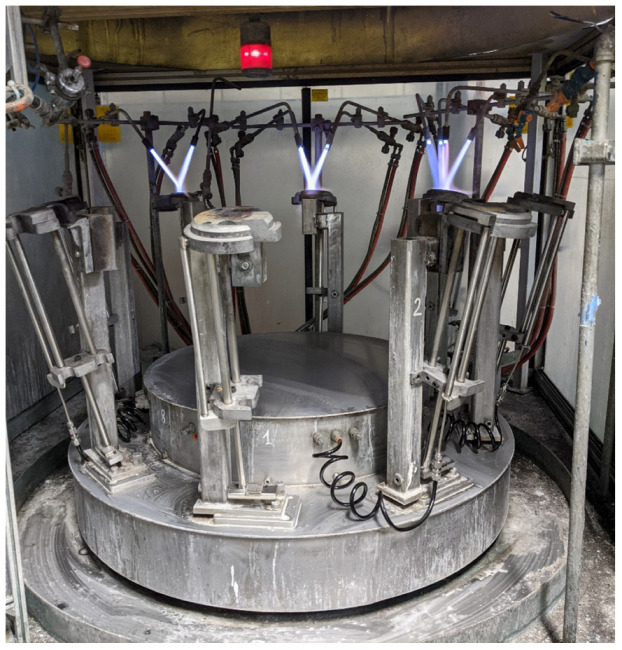
Automatic Brazer with the gas burners on.

The research was conducted on a production line that formed a key process in the manufacture of air conditioning units. The machine under study is involved in the production of pressurised containers responsible for condensing the refrigerant gasses in air conditioning process, known formally as an accumulator. Every air conditioning unit produced in the factory requires an accumulator unit; therefore, the process is an essential part in the assembly line. It was pertinent to study the usage of this machine in order to both capture the capacity usage and the operational costs associated. One part of the assembly of the accumulator unit is performed by the Automatic Brazer, shown in Figure 4. This machine brazes copper pipes onto a steel cap of the accumulator shell. This is one of many essential processes in the assembly of the accumulator unit. 

The Automatic Brazer is a piece of legacy equipment with little control and automation. The machine is comprised of a rotating table with bespoke jig fixtures that hold the subassembly parts in place while being brazed. The table has a total of eight jigs for processing multiple subassemblies at a time. The operator fits the subassembly into the jig fixture while the table is stationary. The table then rotates, moving the subassembly into the next position where flux paste is applied to the braze joint. After the flux is applied, the table is rotated and the subassembly is put into a buffer position. The table is then rotated again, and the part moves into an open flame that heats the metals and forms the braze joint. The next two positions are then also open flame for brazing. After the braze is performed, the penultimate position is the nitrogen purge. The purge performs a crucial process, preventing the fresh braze from oxidising. After being purged, the now fully assembled part rests in the final station and is cooled with compressed air. The unit is then removed after returning to its original position’ or if clearer ‘After returning to its original position, the unit is removed. 

In regular practice, the rotation of the table is automatic and controlled by the PLC. Therefore, the operator of the machine is expected to process the subassembly within an allotted period. If not done within the certain period, the table will have an empty position and waste will occur as the PLC does not turn off the gas supplies if no part is detected. 

The machine is supplied with four gasses to perform the brazing operation. The brazing process is performed with an open flame using the combustion of natural gas and oxygen. These gases are mixed and ignited, powering a total of three burner stations. As detailed above, the braze is protected from oxidisation using a purge of nitrogen gas at one station. To power the movement of the machine table, compressed air is used as an actuator. Compressed air is also used for cooling the unit before removal. The machine has three states: off, idle, and auto. While off, the machine has no gas burners lit and no gas should be consumed. In practice, a small amount of gas is consumed due to leakage. While idle, the burners are lit, and the oxygen and natural gas are consumed. In auto mode, the machine is performing the process to produce parts and therefore all four gasses are consumed. Figure 4 shows a photograph of the Automatic Brazer with the gas burners on.

### 3.2. Investigation

During observations, it became evident that the machine spends considerable time in auto mode, despite not being in use by an operator. This was typically due to production scheduling. Before the Automatic Brazer is used for production, a start-up procedure must be followed. The machine must be allowed to warm up for roughly 15 min before processing a subassembly. Production management often do not know when parts will become available. Therefore, to always be ready for production, the device is left in a ready state. While this simplifies the ability to produce parts, it is an inefficient use of resources.

Instead, the coordination of product flow through the entire process should be better communicated. Extended periods of inactivity should be known and taken advantage of to save on resources and reduce operational costs. M-ACE is currently suffering an increase in production costs due to a price spike in the energy market, where global supply chain issues have increased the cost of the industrial gasses used in this process, for example, nondomestic natural gas prices increased on average by 33% [28]. For M-ACE, monthly natural gas bills have tripled compared to the previous year. Cost reduction, combined with green initiatives pledges the company has undertaken in the past year, provides a large incentive to reduce the amount of waste in the production process.

### 3.3. Data Collection Requirement

With the requirement for data collection in SFs comes storage and connectivity. The data from the machine must be sent over a network to a database for later analysis. Being able to record information in real time also provides the ability to feedback the status of the machine to production management. While implementing these two new features to the manufacturing process, we refer to the RAMI 4.0 to ensure that any new system is compliant with the best practices for I4.0. Two main considerations had to be taken into account: the robustness of data storage and security of the network. 

To move towards a SF is to become a data orientated business. Therefore, it is of the upmost importance that the data the business relies upon is accessible and robust to loss. With a near 24/7 production cycle, the stoppage of the production line is extremely costly. It is not acceptable for the production process to be interrupted due to the inability to access the required data. Any solution for I4.0 must therefore make data available and backed up to ensure that no data loss occurs. 

The requirement for a wider IP network introduces a new emerging risk in I4.0—industrial cyber-attack. Increasing the number of devices on the network increases the attack surface for malicious actors [29]. Industrial Internet of Things (IIoT) devices connect to a network in much the same way IT devices do, however there are key differences in the risk they present in terms of cybersecurity [30]. IIoT devices are embedded systems that are provided with communication functions as an additional feature. 

The processing power and operating system are much more limited than that of a typical IT asset. This creates a problem for the existing IT management as these devices cannot be protected from threats in the same way. IT services and end user devices are all protected from threats with dedicated cybersecurity software that is executed natively on the operating system. The same software cannot be executed on embedded systems. Therefore, the protection of IIoT devices must be assured through external hardware and software. 

## 4. Solution

To monitor and capture the production performance of the Automatic Brazer, the solution required methods to record live data from the machine itself. As discussed above, one of the main issues with the machine is the legacy control equipment. To overcome this issue smart IIoT sensors were retrofitted to the machine. Installing sensors with modern communication protocols allowed for data to be collected to meet the RAMI 4.0 model specification. This also resolved the issue of the poorly maintained control software. Instead of making changes in the existing control software running on the PLC, new sensors gathered data independently of the PLC. To capture the machines efficiency sensors were installed to record the number of units processed and the amount of gas consumed.

### 4.1. Sensor Selection

For the purposes of capturing gas flow rates, IFM IO-Link industrial flow sensors were selected. The sensors operate by connecting to an IO-Link Master module, which provides each individual sensors with both power and communications. The Master module acts as the IIoT device where multiple Ethernet ports can be connected to a standard or industrial IP Network. The Master module reads the data from the slave sensors and packages the data into JavaScript Object Notation (JSON) objects which can be read through a HTTP server hosted on the Master. This methodology provides a simple method to gather data over a network. No additional PLC system was required to gather data, saving on cost, and the sensors themselves were relatively inexpensive compared to competitors.

IFM flow meters were selected for measuring all the gasses under investigation. This made standardisation and spare parts replacement more cost effective. However, in this application, due to the Dangerous Substances and Explosive Atmospheres Regulations (DSEAR) assessment of the area, the flow sensor could not be used on the natural gas pipeline [31]. DSEAR stipulates that areas at risk of explosion must undergo an assessment to categorise the area into a zone rating. This then determines what type of equipment can be used in the assigned zone. The natural gas pipeline itself was categorised as a zone 2, which requires equipment to be rated for use in explosive areas. The solution was to approximate the use of natural gas using the oxygen consumption as oxygen was only consumed together with natural gas. Controls to mix the oxygen and natural gas were fixed; therefore, the consumption of natural gas was assumed proportional to the use of oxygen, based on a defined mix ratio.

To evaluate the utility of the gas use measurement, it was also necessary to know when the machine was operating and producing units. As previously discussed, this was not possible using the existing control system; however, retrofitting a separate data logger was able to resolve this issue. Existing sensors connected to the PLC determined when the table was rotated and if a part was present. From those signals, it is possible to determine how many parts had been processed. To record these signals and collect the data to a database, a Raspberry Pi single board computer (SBC) was installed. 

The Raspberry Pi offered a cost effective and flexible solution to gather data from the legacy PLC. With the use of a logic level convertor, the industrial 24 V signals from the PLC could be stepped down to 5 V and input to the Raspberry Pi GPIO. This methodology provided a way to gather data from the existing system without changing the control software of the commissioned machine. This retrofitting solution once again spared the labour-intensive process of making changes to the existing PLC control software. 

The Raspberry Pi provided access to a wide platform of OSS packages. The data collected via the GPIO could be manipulated in Python and sent to the central database using the ethernet connectivity over the installed network. Having access to the Python programming language meant that OSS database drivers were widely supported, and most database platforms could be supported. 

**Figure 5:**
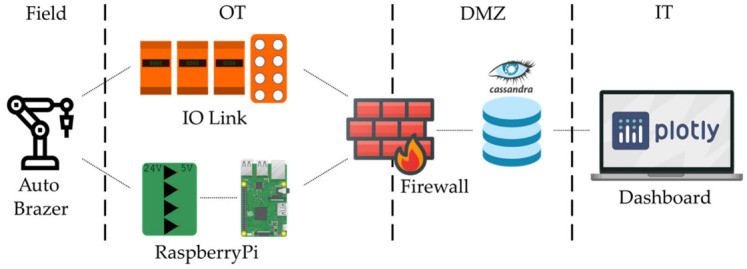
Data flow from Field Machine to End User. Demilitarised zone (DMZ).

Figure 5 illustrates the installed data collection solution. The figure shows the flow of data from the field device to the end user on the IT enterprise network. Process information and status of the Automatic Brazer was collected by the Raspberry Pi and the IO Link Master. Both these devices were provided with ethernet connectivity to a Firewall device. This device provided the necessary cybersecurity solution, as well as the essential routing function to the demilitarised zone (DMZ) between the OT and IT networks; also located in the DMZ was the OSS Cassandra database server for storing the SF data. Having the database located in the DMZ allowed access from both OT and IT devices. The end user would access the data about the field device through a web browser dashboard using their company PC on the IT network.

### 4.2. Cybersecurity 

To protect the M-ACE network from risk, all data collection devices were protected with cybersecurity measures, i.e., a firewall. The TrendMicro EdgeFire Next Generation Firewall (NGFW) was selected for this application. This NGFW was designed for industrial protocols and offered layer 2 through to layer 5 inspection [15]. Proprietary protocols were examined for abnormalities while in transit through the device. If packets were deemed to be suspicious or break user specified rules, they were blocked, and an alert was logged to central management system.

For the Automatic Brazer data collection, only the essential protocols were enabled to pass through the NGFW; all other traffic was blocked. A whitelist of allowed devices was also hardcoded into the device to prevent any foreign devices from connecting to the network from the shop floor. This meant that only the Raspberry Pi and the IO Link Master were able to connect to the network. By only allowing essential traffic and known devices through onto the IT network, the NGFW protected the business network from any security risks from the OT network.

### 4.3. Data Modelling and Database 

Industry 4.0 is driven by data. The continuous operation of production therefore becomes dependent on access to databases and data streams. The stability and availability of the storage of data is of the upmost importance to a digital factory. SFs must not only optimise the availability of their machines but also of their databases. In this research, the OSS Apache Cassandra Database was selected as the supporting data storage for its superior Availability and Scalability [32,33]. For these features, Cassandra has become a popular choice for well known internet technology and social networks companies that have big data solutions [34,35,36].

I4.0 manufacturing works towards a 24/7 production cycle. Manufacturing processes are now dependent on data. SF Databases must therefore prioritise availability above the other two constraints of the Consistency Availability Partition (CAP) theorem [32,37]. Unplanned production downtime due to database failure is unacceptable.

Cassandra can achieve the high availability through its distributed and decentralised architecture [33]. The database creates distribution by operating across multiple machines. Each device forms an individual node in a cluster of Cassandra instances which all work together to form the database. Further redundancy can be implemented by creating a duplicate of the cluster. These clusters can be spread across different physical locations to optimise performance, as well as introducing physical redundancy. The risk of environmental damage or power loss are mitigated by having the database exist in different physical locations. 

Cassandra achieves decentralisation through its no master topology. Each node in the cluster functions identically. Therefore, there is no permanent master node that coordinates the function of the database. With this topology, there is no single point of failure. If a node is lost, the database immediately redirects queries to an alternative node with no loss in performance.

SFs are a rich source of big data. Capturing data from shop floor processes is a high volume and high velocity endeavour. To cope with such a problem, the supporting database must be able to scale with the source. As more assets are added to the system and the historical records grow with time, the volume and velocity of the data would increase. A traditional relational database would struggle to keep up with the forever growing demand for data. Cassandra is able to solve this problem with horizontal scalability [32,33].

With traditional databases, when storage capacity is reached the database needs to be scaled vertically [38]. This is achieved by increasing the specifications of the host machine’s storage, memory, and processing power. In Cassandra, if more memory is required, the database can be scaled horizontally by introducing additional nodes. Each node adds a proportional amount of storage. When a new node is added the load is automatically balanced. The Cassandra service can redistribute the storage load without refactoring the database, thus making the database extremely flexible and scalable.

**Figure 6:**
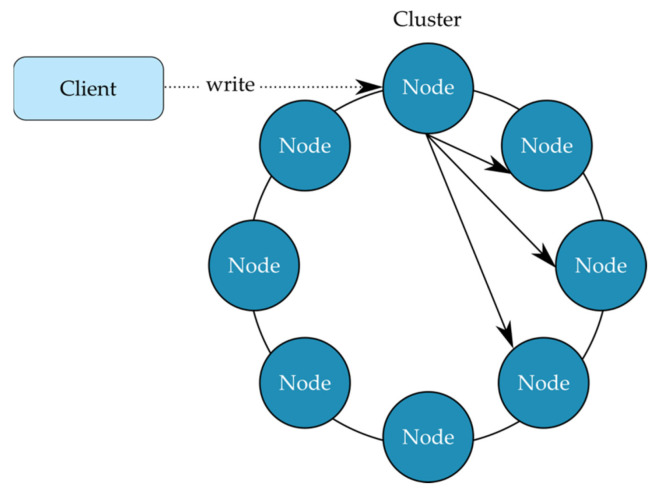
Cluster of Nodes in a Cassandra Cluster.

Figure 6 illustrates the distributed architecture of Cassandra. The example shows multiple nodes that together form a single cluster in a datacenter. When data is written to the database, a replication factor (RF) is specified. This RF determines how many replicas of the data should exist in the cluster. In this example a RF of four is used. The replication process is transparent to the client as they only need to communicate with a single node in the cluster. The Cassandra services perform the replication in the background, spreading the saved data to a further three nodes in the cluster. This provides the high availability in that three out of four nodes in a cluster could be down and the data could still be retrieved. Further redundancy can be implemented by duplicating clusters to another datacenter.

Table 1 shows an excerpt of real data from the database measuring the live flow rate. The timestamp column allows for data queries to be precise, down to the millisecond. The value, unit and type columns allow for the data to be interpreted from a single query.

### 4.4. Dashboarding 

The data analysis function of the SF solution was delivered via a web dashboard. The open source Python library Dash (Plotly) was used to create an interactive and modern platform. The dashboard served a dual purpose, to both display the live status of the machine and historical performance of production.

The dashboard being browser-based allowed for any user with authentication to access the platform without any installation of further software onto the IT enterprise equipment. This greatly simplified deployment and update management since changes did not need to be made of the end user device.

**Figure 7:**
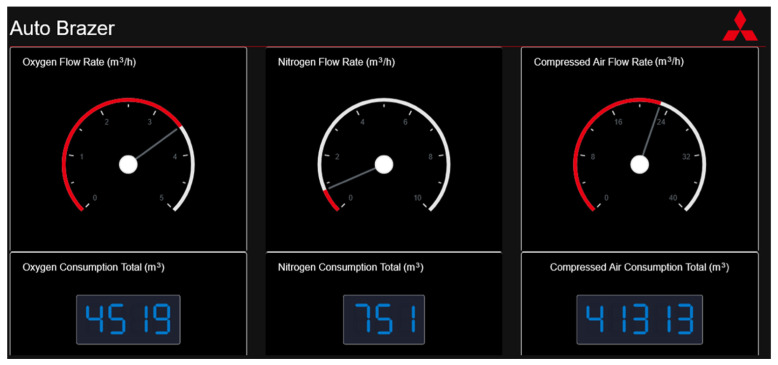
Python Web Dash with live machine data.

The live status of the flow rate is displayed via virtual gauges, allowing production managers to monitor the values and view the current state of the machine. Figure 7 shows the Python dashboard in use, the three gauges show the live flow rate, and a numeric indicator shows the total gas consumption since installation (oxygen, nitrogen and compressed air).

**Figure 8:**
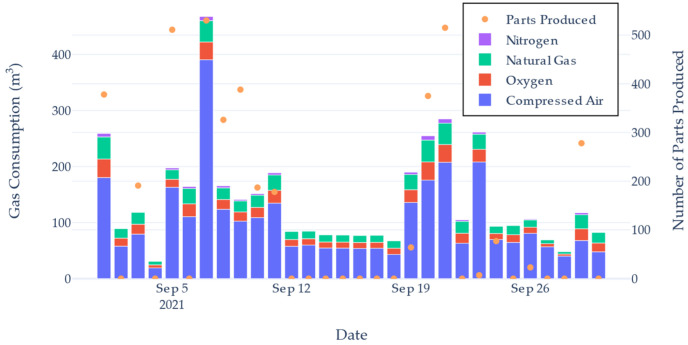
Gas Consumption Total and Part Production Total plot.

Historical data can be inspected using a date range picker. This allows the user to inspect gas usage and production performance over a specific period of interest. Figure 8 shows a histogram of the total gas consumption and a scatter plot of the number of units made. This graph was able to highlight the varying amount of gas used compared to shifts making a similar number of units. The dashboard also plotted a trend of two bespoke *KPI* calculations used to indicate the degree of waste for a production cycle.

Reviewing the data recorded revealed lengthy and irregular intervals of inactivity in production while utilities were still being consumed. This indicated that the machine could have either been better utilised by producing units during these periods, or could have been shutdown to save on resources.

To highlight this inefficiency, two *KPIs* were created based on the productivity of the machine versus the consumption of utilities. The values were calculated on a daily rate, showing the effectiveness of use per day. These values could be plotted live to show the performance on the current day. The first *KPI* was calculated as the ratio of the number of parts produced to the total volume of the gasses consumed that day, shown in (1):

(1) $$KPI_{1}=\frac{n_{units}}{T}$$

where *nunits* is the number of units processed and T is the total volume of gasses consumed (units/m^3^).

**Figure 9:**
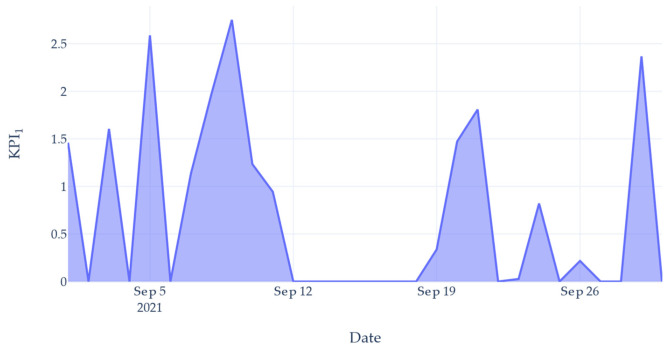
*KPI*_1_—parts per cubic meter of gas.

Figure 9 shows a plot of this $KPI_{1}$ value for the same date range selected in Figure 8. This provided an indication as to how efficient the gas use was on that day. Using the time standard for producing a unit and the nominal gas flow rate, a value of 1.36 units/m^3^ of gas was determined to be satisfactory. Days reporting below this figure were deemed to be inefficient and wasteful. However, $KPI_{1}$ alone was not enough to highlight the level of waste occurring. On days where 0 units were produced, the gas usage is hidden as the value would always be 0. This meant even with heavier gas usage, the *KPI*_1_ value would not change.

To fully capture the level of waste, a second *KPI* was created. This indicator was designed to show the significant waste on days where a small number of units were produced by allowing the value to become a negative number. *KPI*_2_ was calculated by using (2):

(2) $$KPI_{2}=log\left(\frac{n_{units}+1}{T}\right)\;$$

where *n_units_* is the number of units processed and *T* is the total volume of gasses consumed (units/m^3^).

Adding a value of 1 to the numerator ensured *KPI*_2_ was a real number. On days where the number of units produced was low, but the gas use was high, *KPI*_2_ would be below zero to reflect inefficient gas use. A larger negative number would indicate a more severe waste.

**Figure 10:**
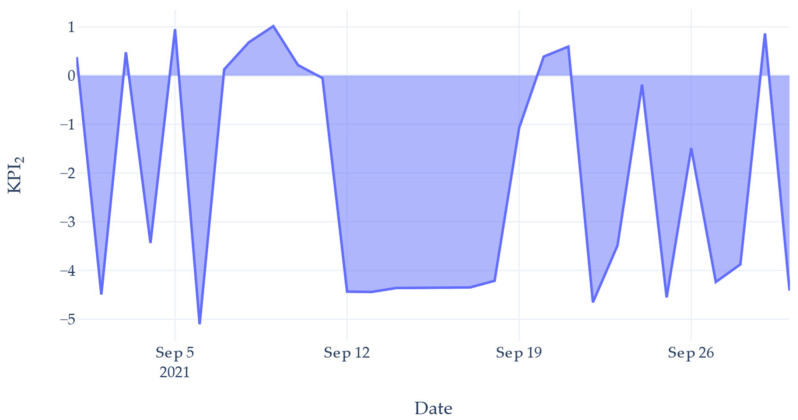
*KPI*_2_—gas consumption effectiveness.

Figure 10 shows the calculated *KPI*_2_ value for the same data presented in Figure 8. The graph shows that even on days where units were produced, a large degree of waste incurred. This was due to the machine being left idle. Had the machine been shut down for these inactive periods, the *KPI*_2_ value would increase. Referring to Figure 8, comparing the days 7th and 21st, both days had produced a similar number of units, yet the 7th consumed considerably more gas. This difference can be seen in Figure 10, where the 7th had a *KPI*_2_ value close to zero, yet the 21st is close to 1. *KPI*_2_ provides a quantitative value for the level of waste occurring on the Automatic Brazer. Production management can interpret this value as an indication of how efficient their operation was.

## 5. Outcome 

### 5.1. Waste Calculation 

To calculate the total waste produced over the studied period, the timeseries data from the Raspberry Pi was used as a mask on the gas consumption data. Each table rotation of the Automatic Brazer was recorded into the database logging a binary value, 1 for part present and 0 for empty. Each part present value recorded provides an indication of productivity. During these periods, it can be deduced that the machine is in its desired operation; on and producing parts. All other times, including when the machine records an empty table rotation and when the machine is completely off, is non-productive time. Gas consumed during this non-productive time window is therefore wasted. 

The window size of the mask was determined by the warm-up time. Considering it is not justified to turn of the machine for a period shorter than it would take to warm-up, the window size was selected to be double the length of the warm-up time. This gave a reasonable leniency for production to leave the machine on in order to resume after a period of inactivity. 

The parts produced time series data were resampled into bins of 30 min. If a unit was processed at any time during the window, the mask was given a value of 0 to mark the period as productive. All other windows were given a value of 1 to mark a period of inactivity. Multiplying the result of the mask with gas consumption data provided an extraction of all total waste. 

**Figure 11:**
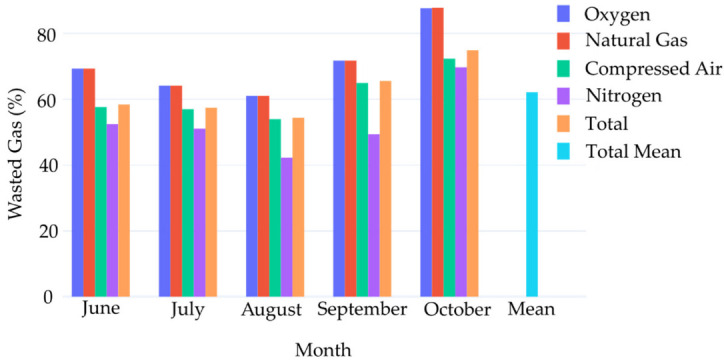
Percentage of waste by gas type.

Figure 11 shows the percentage of waste for each gas for each month of recorded data. Since the beginning of recorded data, 62.1% of all gas consumed was waste. This shows the large potential for waste and cost reduction from the automatic brazing process alone. By examining the data as a time series, it is possible to extract exactly when the machine is unproductive. Instead of using monthly consumption totals, it is possible to more accurately calculate what amount of resources should be required to run production.

### 5.2. Production Feedback

To act on the finding of this research, it was necessary to feedback to the production management. The *KPI* values provided a way for management to view the past and current performance; however, this only provided an indication of waste that has already occurred and administers no active control. 

To create an active response, an automated shutdown of the machine would be scheduled. If the machine was detected to be idle for more than 30 min, a warning email would be sent to the shift manager informing them that the machine would be turned off. If the machine was required to remain on, an email response was sent back from the production manager to cancel the shutdown. They would also be given the opportunity to label the reason for the period of inactivity, e.g., waiting for parts, waiting for labour, maintenance issues. This would help further identify issues with production operations. Having an active control over the machine ensured that the identified waste would be eliminated without the need for human intervention. 

## 6. Conclusions

This research has found that with real time data capture of production performance, it is possible to identify and eliminate wasteful practices. Instead of looking only at a machines production capacity in terms of units, the ratio between the utilities consumed should also be considered. With data gathered as a time series, it is possible to specifically identify periods of inactivity. This analysis can then form a feedback loop to the production operators to prevent further waste. 

The current energy market and green initiatives make waste reduction a great interest to manufacturers. This data collection model forms a part of the pilot program with an intention to expand to the wider factory under study. Using OSS has helped implement an I4.0 system with a small investment to capture the necessary data. With that data, this research has been able to find opportunities to reduce operating costs. 

The need for real time data collection has driven the design of a secure and reliable OT network. The delivered IIoT system was designed to meet the current industry standards using the RAMI 4.0 model. The solution has delivered a robust data collection method, which will ensure data is always available to the production operators. The system has been integrated into the business with security built in to protect and cooperate with the existing enterprise IT networks. 

The implemented solution was able to identify that 62.1% of all gas consumed in the brazing process was waste. With automated feedback, the system was able to alert production management to identify the source of waste. Although performed on a single process, the methods presented are a general template for the other machines in the factory. By comparing the number of units to the amount of utilities consumed, it is possible to achieve a more cost focused measure of production efficiency. These methods will be applied to further machines to achieve more waste reduction across the factory in future works. 

## Figures and Tables

**Table 1:** Excerpt from *data_by_poll_sensor* table in Cassandra.

Sensor ID	Month	Timestamp	Value	Unit	Type
FLOW0011	1 August 2021	1 August 2021 20:30:21:678+001	2.3	m^3^/h	Oxygen Flow Sensor
FLOW0011	1 August 2021	1 August 2021 20:29:51:679+001	2.3	m^3^/h	Oxygen Flow Sensor
FLOW0011	1 August 2021	1 August 2021 20:29:21:682+001	2.4	m^3^/h	Oxygen Flow Sensor
FLOW0011	1 August 2021	1 August 2021 20:28:51:686+001	2.4	m^3^/h	Oxygen Flow Sensor

## Data Availability

The data used in this research is private due to the M-ACE confidentiality policy.

## Abbreviations

IIoT, Industiral Internet of Things; OT, operational technology; KPI, Key Performance Indicator; I4.0, Industry 4.0; IoT, Internet of Things; M-ACE, Mitsubishi Air Conditioning Europe; KTP, Knowledge Transfer Partnership; GCU, Glasgow Caledonian University; ROI, Return on Investment; MQTT, Message Queue Telemetry Transport; OPC UA, Open Platform Communications Unified Architecture; PLC, Programmable Logic Controller; RAMI 4.0, Reference Architecture Model Industrie 4.0; OSS, Open Source Software; API, Application Programming Interface; OS, Operating System; OSH, Open Source Hardware; IPC, Industrial Personal Computer; JSON, JavaScript Object Notation; DSEAR, Dangerous Substances and Explosive Atmospheres; SBC, Single Board Computer; DMZ, Demilitarised Zone; NGFW, Next Generation Firewall; CAP, Consistency Availability and Partition; RF, Replication Factor.
